# Northern Hemisphere 2025/26 influenza vaccine effectiveness during months with influenza A(H3N2) subclade K predominance— Suzhou, Eastern China

**DOI:** 10.1016/j.ijid.2026.108552

**Published:** 2026-03-15

**Authors:** Pengwei Cui, Suizan Zhou, Anna N. Chard, Junrong Wang, Kathrine R. Tan, Ying Song, Yuanyuan Pang, Yuanyuan Zhang, Huiyan Yu, Zefeng Dong, Zhiyuan Zhu, Yayun Tan, Haibing Yang, Haitao Wang, Xiaokun Yang, Zhibin Peng, Kathryn E. Lafond, Eduardo Azziz-Baumgartner, Liling Chen

**Affiliations:** 1 Suzhou Municipal Center for Disease Control and Prevention, Suzhou, Jiangsu Province, China; 2 Influenza Division, National Center for Immunization and Respiratory Diseases, CDC, Atlanta, GA, USA; 3 Jiangsu Provincial Center for Disease Control and Prevention, Nanjing, Jiangsu Province, China; 4 Suzhou Health Information Center, Suzhou, Jiangsu Province, China; 5 Division of Infectious Disease, Chinese Center for Disease Control and Prevention, Beijing, China

**Keywords:** Vaccine effectiveness, Northern hemisphere, Subclade K, Influenza

## Abstract

During this 2025/26 Northern Hemisphere influenza season, the predominant influenza A(H3N2) subclade K virus has binding site mutations and postvaccine sera reduced inhibition, raising concerns about antigenic differences with available vaccines. During November 2025–January 2026, 8,455 patients with influenza-like illness (ILI) and 4,479 patients with severe acute respiratory illness (SARI) were identified through 25 sentinel surveillance hospitals in Suzhou City, Jiangsu Province, China. Among enrolled ILI and SARI patients, 4,032 (47.7%) and 697 (15.6%) were influenza positive by RT-PCR, respectively. Of all influenza positives, four were influenza B Victoria and three were influenza A(H1N1)pdm09; all remaining positives were influenza A(H3N2). Among gene sequenced viruses isolated from 44 randomly-selected positive samples during the study period, 95% were influenza A(H3N2) subclade K. Generalized linear regression models indicated Northern Hemisphere 2025/26 influenza vaccines, including A/Croatia/10136RV/2023 (H3N2)-like viruses, reduced risk for influenza-associated outpatient visits by approximately one-half during the November 2025–January 2026 subclade K dominant months. We did not observe statistical significance, although the point estimate suggested a possible one-fifth reduction in influenza-associated hospitalizations. Increasing seasonal influenza vaccine coverage could relieve stress on the medical system during respiratory disease season.

## Introduction

Influenza vaccines are most effective at preventing medically attended influenza illnesses during seasons when their formulation includes antigens similar to the predominantly circulating influenza viruses in communities. During this 2025/26 Northern Hemisphere influenza season, the predominant influenza A(H3N2) subclade K virus has binding site mutations and postvaccine sera reduced inhibition, raising concerns about antigenic differences with available vaccines [1, 2]. Using data from influenza-like-illness (ILI) and severe acute respiratory illness (SARI) surveillance networks in Suzhou City, Jiangsu Province, China, we estimated Northern Hemisphere influenza vaccine effectiveness (VE) during a period dominated by influenza A(H3N2) subclade K circulation.

## Material and methods

During November 2025-January 2026, we conducted ILI and SARI sentinel surveillance across 25 hospitals in Suzhou. ILI and SARI cases were identified using systematic sampling based on national protocols [3]. At each sentinel site, medical staff systematically selected the first 5–10 ILI and SARI patients per day for respiratory specimen collection to ensure consistent daily representation. ILI was defined as acute respiratory infection with a body temperature of 38 °C or higher accompanied by either a cough or a sore throat in the outpatient setting, and SARI was defined as an ILI case requiring hospitalization. Patients were tested for influenza viruses by reverse-transcription-polymerase chain reaction (RT-PCR) and typed and subtyped in reference laboratories. Each week, 3–4 influenza positive specimens with CT values below 32, were randomly selected for genetic sequencing. Vaccination status was determined using vaccination registries. Only World Health Organization recommended egg-based Northern Hemisphere influenza vaccine formulations were administrated in Suzhou during the 2025/26 season; these contained A/Victoria/4897/2022 (H1N1)pdm09-like, A/Croatia/10136RV/2023 (H3N2)-like, and B/Austria/1,359,417/2021-like virus antigens [4].

VE against influenza-associated outpatient visits and hospitalization was estimated using a test-negative case-control design. Case-patients were those who received a positive influenza RT-PCR test result; control-patients were those who received a negative influenza RT-PCR test result. Patients who received at least one 2025/26 influenza vaccine dose ≥14 days before symptom onset were considered vaccinated against influenza; those not vaccinated before symptom onset were considered unvaccinated. Patients who were vaccinated < 14 days before symptom onset were excluded.

VE was estimated using generalized linear regression models that compared the odds of influenza vaccination between case-patients and control-patients, adjusting for sex, age years (fit as a cubic spline with five knots), and symptom onset date (fit as a cubic spline with five knots) [5]. VE was calculated as (1- adjusted odds ratio) × 100%. In addition to the overall estimate, which includes all vaccine-eligible (i.e., aged ≥6 months) ILI and SARI patients, VE estimates and 95% confidence intervals (CIs) were stratified by selected recommended influenza vaccination priority groups in China, including young children (aged 6 months–4 years), school-aged children (aged 6–17 years), and older adults (aged ≥60 years) [6]. R statistical software (version 4.5.1) was used for analysis.

## Results

During the study period, 8,455 patients with ILI and 4,479 patients with SARI were included in the analysis. Among ILI and SARI patients, 4,032 (47.7%) and 697 (15.6%) were influenza positive, respectively (Table 1). Over 99% of 4,729 influenza positives were due to influenza A(H3N2) infection, with only four influenza B Victoria infections (three ILI and one SARI) and three influenza A(H1N1)pdm09 infections (all ILI). Among gene sequenced viruses isolated from 44 randomly-selected positive samples during the study period, 95% were influenza A(H3N2) subclade K. Among the 4,032 ILI case-patients, 98 (2.4%) had received at least one 2025/26 seasonal influenza vaccine, compared with 142 (3.2%) of 4,423 ILI control-patients. Of the 697 SARI case-patients identified, 19 (2.7%) had received at least one 2025/26 seasonal influenza vaccine compared with 104 (2.7%) of 3,782 SARI control-patients.

Among patients with ILI, VE against influenza-associated outpatient illness with any influenza virus was 48% (95% CI: 31–61) (Table 1). VE against influenza-associated outpatient illness with any influenza virus was 58% (95% CI: −9 to 87) among young children, 45% (95% CI: 24–61) among school-aged children, and 49% (95% CI: −171 to 97) among older adults.

Among patients with SARI, VE against influenza-associated hospitalization with any influenza virus was 22% (95% CI: −29 to 55). VE against influenza-associated hospitalization with any influenza virus was 36% (95% CI: −53 to 79) among young children, 9% (95% CI: −103 to 62) among school-aged children, and 44% (95% CI: −196 to 97) among older adults.

## Discussion

Despite evidence of subclade K binding site mutations, Northern Hemisphere 2025/26 influenza vaccines, including A/Croatia/10136RV/2023 (H3N2)-like viruses, reduced risk for influenza-associated outpatient visits by approximately one-half during the November 2025-January 2026 subclade K dominant months. This analysis was underpowered for hospitalization outcomes, and we did not observe statistically significant VE against influenza-associated hospitalization. Our estimates suggested that influenza vaccination may reduce influenza-associated hospitalizations by approximately one-fifth, but given the imprecision of this estimate it should be interpreted with caution. Our estimates are slightly lower than interim findings from the United Kingdom and European Union, particularly among pediatric populations [1, 7].

The VE estimates against ILI and SARI reflected the overall effect on our study participants. The largest proportion of ILI patients were school-aged, which likely reflects age-specific differences in healthcare-seeking behavior. Similar with the population-based influenza notifiable disease reporting, children in this age group with ILI/SARI were more likely to seek medical care and subsequently be captured by surveillance. Notably, school-aged children are also a priority population under the national influenza vaccination policy in China [6]. Although we adjusted for age using multivariable generalized linear regression and reported VE based on adjusted odds ratios, caution is warranted when generalizing to populations with different age structures.

Increasing seasonal influenza vaccination coverage, especially among groups at increased risk for complications from influenza [8], could prevent medically attended influenza illness and stress on the medical systems during the remainder of the 2025/26 influenza season.

## Figures and Tables

**Table 1:** Interim 2025/26 Northern Hemisphere seasonal influenza vaccine effectiveness against influenza in patients with influenza-like illness and patients with severe acute respiratory illness, Suzhou City, Jiangsu Province, China, November 2025-January 2026.

	No. (column %)	Vaccinated, no (row %)^1^	*p*-value^2^	Positive influenza test result, no (row %)^3^	*p*-value^2^	Case-patients with positive influenza test results^3^		Control-patients with negative influenza test results		Influenza vaccine effectiveness^4^, % (95% CI)
	
						Total, no.	Vaccinated, no. (%)	Total, no.	Vaccinated, no. (%)	

Patients with ILI, total	8455 (65.4)	240 (2.8)	–	4032 (47.7)	–	4032	98 (2.4)	4423	142 (3.2)	48 (31, 61)
Priority vaccination group^5^										
Young children	571 (6.8)	32 (5.6)	<0.001	153 (26.8)	<0.001	153	5 (3.3)	418	27 (6.5)	58 (−9, 87)
School-aged children	3855 (45.6)	161 (4.2)		2491 (64.6)		2491	85 (3.4)	1364	76 (5.6)	45 (24, 61)
Older adults	1147 (13.6)	19 (1.7)		145 (12.6)		145	1 (0.7)	1002	18 (1.8)	49 (−171, 97)
Patients with SARI, total	4479 (34.6)	123 (2.7)	–	697 (15.6)	–	697	19 (2.7)	3782	104 (2.7)	22 (−29, 55)
Priority vaccination group^5^										
Young children	1597 (35.7)	62 (3.9)	<0.001	162 (10.1)	<0.001	162	5 (3.1)	1435	57 (4.0)	36 (−53, 79)
School-aged children	815 (18.2)	32 (3.9)		236 (29.0)		236	9 (3.8)	579	23 (4.0)	9 (−103, 62)
Older adults	1309 (29.2)	13 (1.0)		159 (12.1)		159	1 (0.6)	1150	12 (1.0)	44 (−196, 97)

**Abbreviations:** ILI = influenza-like illness; SARI = severe acute respiratory illness; CI = confidence interval.

1 Patients who received ≥1 dose of the 2025/26 season influenza vaccine ≥14 days before symptom onset were considered vaccinated; patients who did not receive any influenza vaccine during the 2025/26 season by the time of symptom onset were considered unvaccinated. Patients vaccinated 0–13 days before symptom onset were excluded.

2 Pearson’s chi-square test was used to ascertain differences in the number of persons who were vaccinated and unvaccinated against influenza or who received positive and negative influenza test results.

3 Of the positive cases, four were influenza B Victoria and three were influenza A(H1N1)pdm09; all remaining positives were influenza A(H3N2). Reverse-transcription polymerase-chain reaction testing for influenza typing and subtyping was conducted at reference laboratories in Suzhou City.

4 Vaccine effectiveness (VE) was estimated using generalized linear regression models that compared the odds of influenza vaccination between case-patients and control-patients, adjusting for sex, age years (fit as a cubic spline with five knots), and symptom onset date (fit as a cubic spline with five knots). VE was calculated as (1- adjusted odds ratio) × 100%.

5 Young children were defined as those aged 6 months-4 years; school-aged children were defined as 6 years-17 years; older adults were defined as those aged ≥60 years.

## References

[1] Kirsebom FCM, Thompson C, Talts T, Kele B, Whitaker HJ, Andrews N, Early influenza virus characterisation and vaccine effectiveness in England in autumn 2025, a period dominated by influenza A(H3N2) subclade K. Eurosurveillance 2025; 30 (46):2500854. 41267661 10.2807/1560-7917.ES.2025.30.46.2500854 PMC12639273

[2] Zambon M, Hayden FG. Influenza A(H3N2) subclade K virus: threat and response. J Am Med Assoc 2026; 335 (4):307–10. 10.1001/jama.2025.25903 41411120

[3] Chinese Center for Disease Control and Prevention National Influenza Surveillance Technical Guidelines. 2017 Edition; 2018. [accessed 23 February 2026]. https://ivdc.chinacdc.cn/cnic/fascc/201802/P020180202290930853917.pdf

[4] World Health Organization Recommended composition of influenza virus vaccines for use in the 2025–2026 northern hemisphere influenza season. [accessed 23 February 2026] https://www.who.int/publications/m/item/recommended-composition-of-influenza-virus-vaccines-for-use-in-the-2025-2026-nh-influenza-season.

[5] Russ S, Nogareda F, Regan AK, Benedetti E, Pasinovich M, Voto C, Interim effectiveness estimates of 2025 Southern Hemisphere influenza vaccines in preventing influenza-associated outpatient and hospitalized illness - eight Southern Hemisphere countries, March-September 2025. MMWR Morb Mortal Wkly Rep 2025; 74 (36):570–8. 40996960 10.15585/mmwr.mm7436a3 PMC12463193

[6] Chinese Center for Disease Control and Prevention Technical guidelines for the application of seasonal influenza vaccine in China (2025–2026). [accessed 23 February 2026] https://ivdc.chinacdc.cn/cnic/zxhd/202511/P020251103518567286583.pdf.

[7] European Centre for Disease Prevention and Control Early estimates of seasonal influenza vaccine effectiveness against influenza requiring medical attention at primary care level in Europe, week 41 – 49, 2025. [accessed 23 February 2026] https://www.ecdc.europa.eu/en/news-events/early-estimates-seasonal-influenza-vaccine-effectiveness-against-influenza-requiring.

[8] Centers for Disease Control and Prevention People at increased risk for flu complications. [accessed 23 February 2026] https://www.cdc.gov/flu/highrisk/index.htm.

